# Skin Lesion Classification Based on Surface Fractal Dimensions and Statistical Color Cluster Features Using an Ensemble of Machine Learning Techniques

**DOI:** 10.3390/cancers13215256

**Published:** 2021-10-20

**Authors:** Simona Moldovanu, Felicia Anisoara Damian Michis, Keka C. Biswas, Anisia Culea-Florescu, Luminita Moraru

**Affiliations:** 1 Department of Computer Science and Information Technology, Faculty of Automation, Computers, Electrical Engineering and Electronics, Dunarea de Jos University of Galati, 47 Domneasca Str., 800008 Galati, Romania; simona.moldovanu@ugal.ro; 2 The Modelling & Simulation Laboratory, Dunarea de Jos University of Galati, 47 Domneasca Str., 800008 Galati, Romania; felicia.michis@ugal.ro; 3 Department of Chemistry, Physics & Environment, Faculty of Sciences and Environment, Dunarea de Jos University of Galati, 47 Domneasca Str., 800008 Galati, Romania; 4 Department of Biological Sciences, University of Alabama at Huntsville, Huntsville, AL 35899, USA; kcb0015@uah.edu; 5 Department of Electronics and Telecommunications, Faculty of Automation, Computers, Electrical Engineering and Electronics, Dunarea de Jos University of Galati, 47 Domneasca Str., 800008 Galati, Romania; anisia.florescu@ugal.ro

**Keywords:** skin cancer recognition, k-nearest neighbor, Higuchi fractal dimensions, Radial basis function neural network

## Abstract

**Simple Summary:**

This study aimed to investigate the efficacy of implementation of novel skin surface fractal dimension features as an auxiliary diagnostic method for melanoma recognition. We therefore examined the skin lesion classification accuracy of the kNN-CV algorithm and of the proposed Radial basis function neural network model. We found an increased accuracy of classification when the fractal analysis is added to the classical color distribution analysis. Our results indicate that by using a reliable classifier, more opportunities exist to detect timely cancerous skin lesions.

**Abstract:**

(1) Background: An approach for skin cancer recognition and classification by implementation of a novel combination of features and two classifiers, as an auxiliary diagnostic method, is proposed. (2) Methods: The predictions are made by k-nearest neighbor with a 5-fold cross validation algorithm and a neural network model to assist dermatologists in the diagnosis of cancerous skin lesions. As a main contribution, this work proposes a descriptor that combines skin surface fractal dimension and relevant color area features for skin lesion classification purposes. The surface fractal dimension is computed using a 2D generalization of Higuchi’s method. A clustering method allows for the selection of the relevant color distribution in skin lesion images by determining the average percentage of color areas within the nevi and melanoma lesion areas. In a classification stage, the Higuchi fractal dimensions (HFDs) and the color features are classified, separately, using a kNN-CV algorithm. In addition, these features are prototypes for a Radial basis function neural network (RBFNN) classifier. The efficiency of our algorithms was verified by utilizing images belonging to the 7-Point, Med-Node, and PH2 databases; (3) Results: Experimental results show that the accuracy of the proposed RBFNN model in skin cancer classification is 95.42% for 7-Point, 94.71% for Med-Node, and 94.88% for PH2, which are all significantly better than that of the kNN algorithm. (4) Conclusions: 2D Higuchi’s surface fractal features have not been previously used for skin lesion classification purpose. We used fractal features further correlated to color features to create a RBFNN classifier that provides high accuracies of classification.

## 1. Introduction

The increasing number of skin cancer patients has revealed the need for decision support systems that can detect the lesions with high accuracy. In Europe, more than 100,000 melanoma cancers and around 22,000 skin cancers associated to melanoma are reported each year [1]. In Australia, more than 14,000 people with melanoma are reported yearly [2]. In the US, in 2018, more than 91,000 new cases and more than 13,000 deaths were reported [3]. In addition, the number of melanoma cases increases annually: An increase of 225% has been reported in the US over the last 30 years [3,4].

The primary form for melanoma detection is visual clinical examination. Visual inspection is an unaided method and thus a challenging and subjective task with limited accuracy, as it is strongly dependent on the expertise of the individual. A properly trained dermatologist uses various screening methods such as the 7-point checklist (describing the symptoms of an atypical pigment network, grey-blue areas, and atypical vascular patterns, blotches, streaks, irregular dots, and globules) [5], the ABCDE rule (i.e., A is for asymmetry, B is for border irregularity, C represents color variations, D is for the diameter, and E for evolution) [6], and other techniques such as infrared imaging, multispectral imaging, and confocal microscopy [7,8,9,10] for melanoma detection. They serve as adjuncts to physicians and provide a satisfactory performance, but are laborious and prone to human error. The accurate diagnosis of the skin cancer in its early stages may reduce the mortality rate by up to 90% [11,12]. The research on medical image processing has led to the development of artificial intelligence, especially deep learning systems to support the diagnosis of various skin lesions.

The strategy for early diagnosis is recognizing new skin lesions or changes in shape, borders, color, and geometry of existing lesions. Most of the existing methods for automated skin lesions detection from dermoscopy images incorporate the following steps: image preprocessing, segmentation, feature extraction, and classification. Segmentation or lesion border detection refers to the position identification. Manual segmentation is tedious, time-consuming, and susceptible to higher inter- and intra-observer variability due to the great variety of lesion shapes, textures, and colors. In addition, the different imaging conditions disturb both the segmentation and feature extraction processes [13,14]. Skin lesion color is another salient feature for diagnosing melanoma. Certain colors are mostly correlated to melanocytic lesions, including brown or black, shades of tan and occasional patches of red, white, or blue [15,16]. However, the existing variability due to the skin color and presence of hairs or veins may lead to variability in the aspect of the melanoma, in terms of color and texture. Various color features can be extracted from the color channels by searching for certain statistical values, such as color asymmetry or centroid distance. This can also be done by using global and local descriptors. Six shades of color are present in skin lesions, light brown, dark brown, white, red, blue, and black [17], but there are difficulties and discrepancies in color perceptions among dermatologists. It would be beneficial to use an algorithm that can identify the clusters of color that closely match dermatologists’ color perception. The choice of suitable color cluster composition is a major bottleneck in these studies.

The border’s irregularity (i.e., the B feature of the ABCDE rule) can be objectively measured using a fractal dimension. These fractal dimensions are related to the complexity of the shapes and have been used for skin lesion border irregularities characterization [18,19]. Ali et al. [20] proposed a deep learning algorithm to learn and classify border irregularities by viewing them as subtle structures of mole and melanoma images. The box-counting method was employed to estimate the fractal dimension of the irregular outlines. Melanomas have irregular borders and they are anticipated to have a higher fractal dimension compared to regular nevi. A 1D HFD was effectively used to compare the brain EEG signal complexities in experiments carried out on rats. A 2D HFD was proposed in [21] for cells and tissue in plant images and in [22] for histological images for an efficient differentiation of tumors.

Artificial intelligence (AI) tools facilitate substantial improvements in all areas of healthcare (either direct healthcare or drug development and ambient assisted living) from earlier diagnosis, customized treatments, monitoring, and efficient follow-ups. However, deep learning algorithm solutions are still relatively new to clinical applications in term of facilitating and enhancing the clinicians’ work in their skin cancer diagnosis process. AI solutions involve medical visualization above human-level capabilities and have an important impact on the current medical practice of dermatology where the patients are examined by dermoscopy. The goal is to augment certain features to facilitate skin cancer classification that is equal to or better than dermatologists’ performance.

Recently, various types of deep-learning neural network image classifiers were proposed to achieve better diagnosis performance. Although a growing body of research suggests deep learning approaches as the most successful applications in images classification, they are so called “black-box” models with a huge predictive power but whose inference mechanisms are often difficult, if not impossible, to grasp intuitively. This is an important obstacle to implementing deep-learning neural network classifiers in routine medical practice. The computational complexity is also a challenge. In our previous study [23], we showed the feasibility of a feedforward back propagation network algorithm that is based on asymmetry, moment of inertia, and histograms to differentiate between non-melanoma and melanoma. Specifically, 60 different net architectures were trained and tested in a dataset collected from four databases (7-Point, PH2, MED-NODE, and PAD-UFES-20). The model performance had an average accuracy of 96.7% and a mean square error (MSE) of 0.0203.

There have been very few studies about the implementation of the 2D HFD as a new descriptor for the study of skin lesions and as input data for a classifier. In this paper, we expand on our previous study [23] and propose a method to derive and cluster the color features of skin lesions, to derive the fractal dimension of the whole skin lesion image by implementing the 2D Higuchi’s surface fractal dimension, and to use machine learning models to automatically identify benignity or malignancy based on these measurements. The main contributions are as follows:

(i) Determining the representative average percentage of color areas of skin lesions for each considered dataset.
(ii) Proposing a descriptor for the investigation of the skin surface fractal dimensions for the channels in RGB color images, i.e., 2D Higuchi fractal dimension as an objective quantitative.
(iii) Two distinctive machine learning classifiers, namely a kNN-CV algorithm and a RBFNN approach as a non-linear classifier, are implemented to generate the prediction. A dynamic partitioning of data is carried out using the 5-fold cross validation method (CV). These machine learning classifiers belong to different classification paradigms.

To the knowledge of the authors, the proposed techniques are novel and there is room for improvement of the existing methods already presented in the literature.

The rest of this paper has the following structure: Section 2 presents a review of the current diagnostic tools; Section 3 presents the proposed methodology, introduces the features to be used, describes the classifier architectures and implementation details, and discusses the image datasets; Section 4 shows the experimental results and discussions; finally, Section 5 gives some concluding remarks and some future work directions.

## 2. Related Works

Deep learning and neural networks devoted to image segmentation have been increasingly applied to various fields, from satellite imagery and medical scans to cultural heritage preservation and biomechanics [24,25,26,27,28,29,30,31]. Remarkable progress in machine learning methods have been reported for a dermatologist-level skin cancer classification. Here, we summarize the current state-of-the-art developments.

Recently, AI and deep learning techniques have been employed in skin cancer classification. The color distributions of skin lesions have been grouped into six shades of color, which arise due various biological processes. Various AI methods are employed to correlate the melanocytic lesions with certain colors such as brown or black, shades of tan, and occasional patches of red, white, or blue. Khan et al. [16] proposed a hybrid super-feature vector containing textural and color features to realize an intelligent system able to discriminate melanoma from nevus. A support vector machine (SVM) was utilized as a classifier on the DERMIS dataset with 397 skin cancer images. The proposed method achieved 96% accuracy. In another study [32], six different color spaces were used to extract color as a global feature (i.e., mean, standard deviation, variation, and skewness) to classify melanoma and non-melanoma. They reported an accuracy of 90.32%. Seidenari et al. [33] described the colors in melanocytic lesion images by means of the distribution of the 23 color clusters for the RGB images. A significant difference in the presence of the selected color clusters between the nevi and melanoma populations was reported. A sensitivity of 85.5% was reported based on only one parameter (color clusters). A combined approach of deep learning techniques with clinical criteria (color, texture, and shape) for the automated diagnosis of skin lesions is reported in [34]. 

The 1D Higuchi fractal dimension (HFD) [35] is used as a discriminative feature for various classifiers to classify the modulated signals. This fractal dimension shows superior noise immunity despite having a higher computational complexity. A general application of HFD for a planar curve was proposed in [21,22]. This 2D fractal dimension was only used for histological images. Despite the efficacy of fractal dimensions as meaningful features in machine learning applications, there has been limited research using 2D HFD data sequences as a measure of irregularity of skin lesion borders. Čukić et al. [36] used the 1D HFD together with sample entropy to analyze brain neuronal activity in depressive disorders. These two relatively uncorrelated features provided a better classification accuracy for all employed machine learning techniques. The 4- or 8-connectivity are considered in [37] for neighboring data points. The 2D Higuchi fractal dimensions for both greyscale and color images are computed. A very good performance was reported in the case of color images. 

Despite advantages in feature extraction and classification accuracy, a few limitations exist, such as an important variability in the quality of dermoscopy images along with a low resolution that characterizes many of these images. In addition, there is no machine learning technique available yet that can extract and learn the most important and discriminant features from a high dimensional dataset to obtain a higher classification accuracy.

## 3. Proposed Methodology

**Figure 1:**
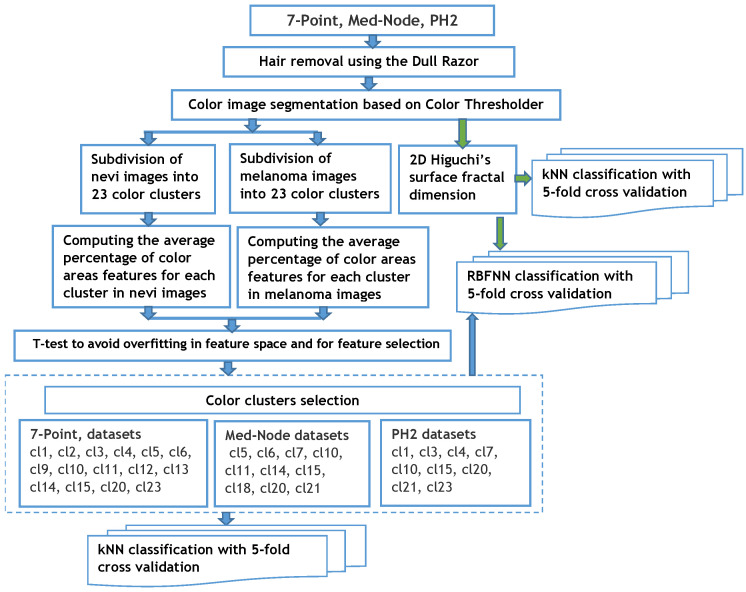
Processing steps for classifying skin lesions.

The proposed method consists of the following primary steps, which can be seen in Figure 1.

**Figure 2:**
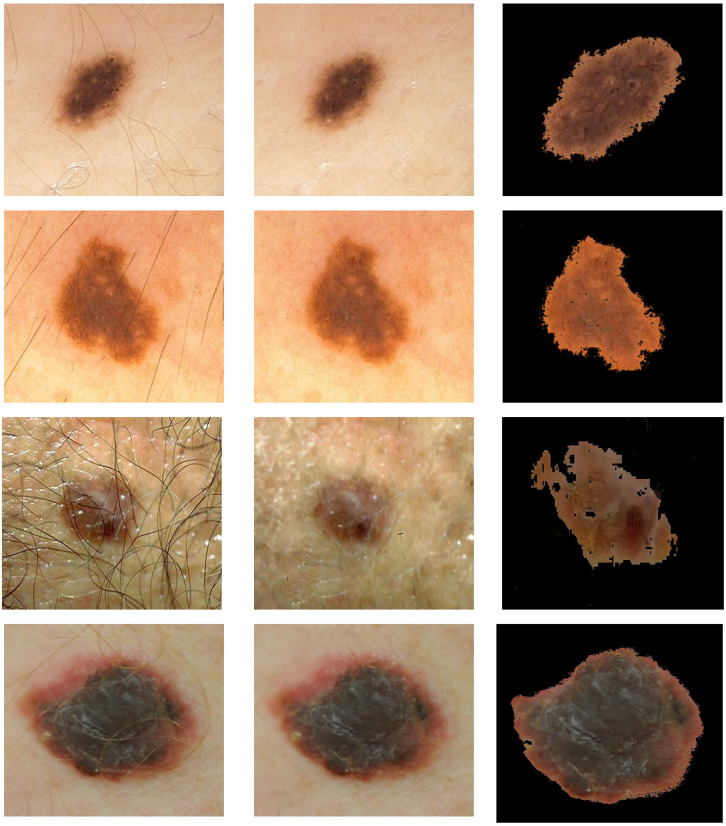
Illustration of pre-processing for hair removal and segmentation. Rows 1 and 2: nevi image (PH2 database). Rows 3 and 4: melanoma image (7-Point database in row 3 and Med-Node database in row 4). First column: original image. Second column: image after the hair is removed. Third column: segmented image. Med-Node contains non-dermoscopic (or simple digital) images acquired via cross-polarized light. Skin lesions in PH2 were imaged via polarized noncontact dermoscopy by using conventional cross-polarized light. Skin lesions in 7-Point were imaged via fluid immersion and non-polarized dermoscopy.

The first step is image pre-processing for noise reduction, hair removal, and image segmentation (Figure 2). 

Then, a clustering method allows for the selection of the relevant color distribution in the melanocytic lesion images and computation of the average percentage of color areas within the nevi and melanoma lesion area. A *t*-test is used for feature selection and to reduce the overfitting in the feature space. In a third step, the fractal dimension of the surface is computed using a 2D generalization of HFD. It computes the HFD of the R, G, and B channel colors in RGB images associated to the skin lesions. Both the color and fractal features are classified, separately, using a kNN algorithm with a 5-fold cross validation method. The same discriminant features are utilized for the classification by the RBFNN approach as those for a non-linear classifier. A performance analysis of these different classification paradigms is performed.

### 3.1. Analysis of Relevant Color Distribution in Melanocytic Lesion Images and Color Clusters Selection

**Figure 3:**
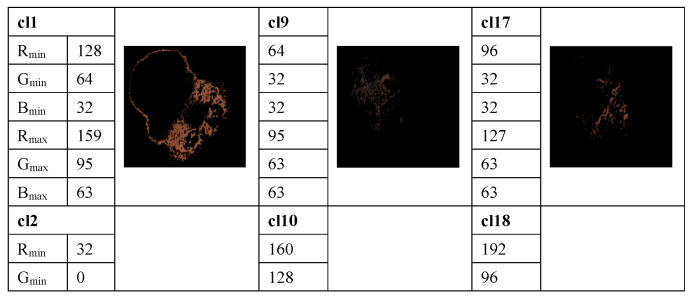

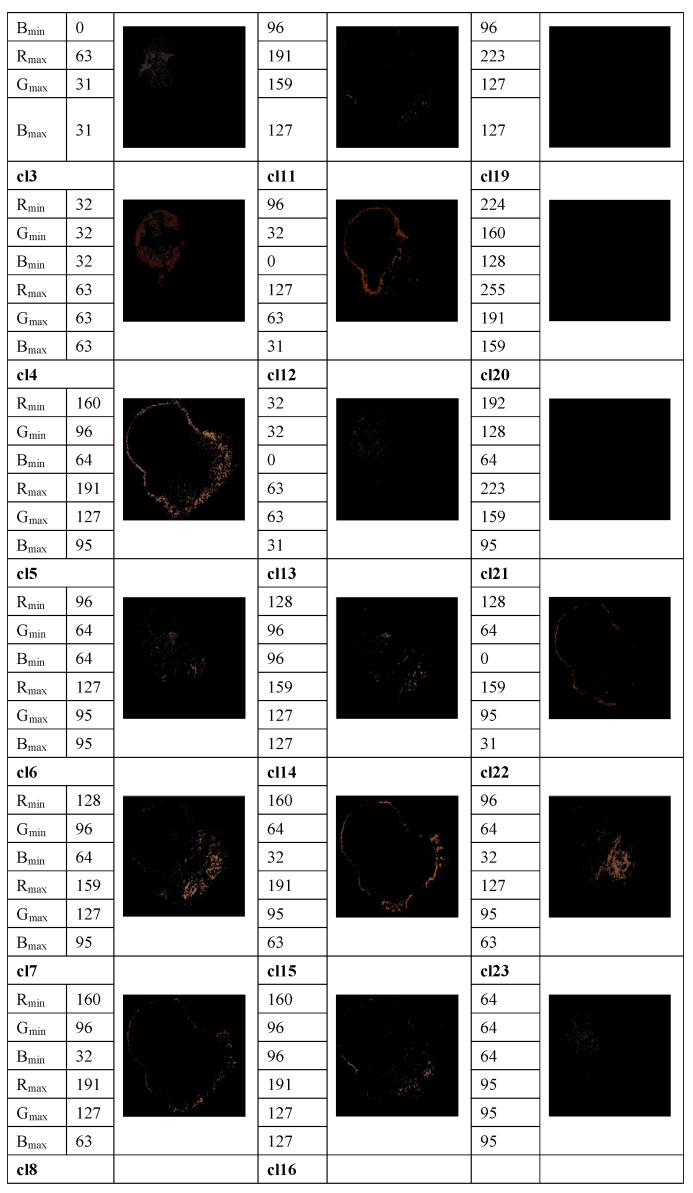

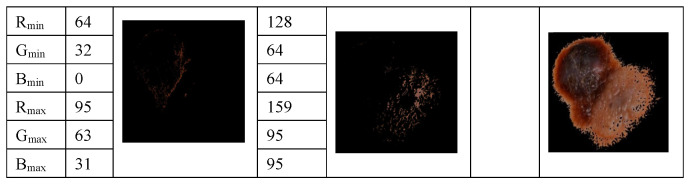
Example of measuring the color content and clustering method to select the relevant color distribution in melanocytic lesion images. 23 color clusters, denoted as cl1, …, cl23, are presented. Data for the minimum and maximum intensity in each R, G, and B channel are indicated for each color cluster. Lower right: the segmented image.

The method proposed in [33] is used to determine the average percentage of color areas within the nevi and melanoma lesion area. For this purpose, we analyze the color histogram of each image associated with melanoma and nevus lesions. A clustering method allows for the selection of the relevant color distribution in melanocytic lesion images. In the color histogram of the lesion, the pixels are mapped to the relative color clusters. Thus, we considered twenty-three color clusters, denoted as cl1, …, cl23, that are characterized by the largest differences between the minimum and maximum intensity in each R, G, and B channel, according to the data provided in [33]. For each image the percentage of each color cluster is computed as the ratio between the number of pixels within the lesion belonging to the specified color cluster and the total pixels of the lesion. These average percentages of color areas represent relevant statistical features for distinction among melanocytic lesions. A feature space is built. However, many features are correlated and the redundant information about the information content of the images worsens the classification performance. To avoid this issue, a *t*-test is used for the feature selection, by removing features with no predictive information. In addition, the overfitting in the feature space is reduced. A value of *p* < 0.05 is considered statistically significant, i.e., the selected samples are different from one another in a statistically significant way. Selected features are universally accepted and used for malignancy detection, but they do not yield satisfactory performance on their own. An example of the color detection, color prototype in the RGB image, and the quantized color clusters after this process is completed are shown in Figure 3.

### 3.2. Higuchi’s Surface Fractal Dimension (HFD)

HFD is proposed as a tool for detecting and classifying skin lesions based on the complexities present in the images. The Higuchi algorithms run from the minimal scale *k =* 1 to the maximal scale *kmax = 8*. A surface *f* in the 3D Euclidean space allows for a graphical representation $\gamma_{ij}=f\left(i,j\right)$, *i* = 1, …, *M, j* = 1, …, *N.* According to [20], $\gamma_{ij}$ denotes the intensity levels of a 2D image and they are the element of a *(M × N)* matrix noted as X. To define the elements of the matrix X, the plane surface *f* is tessellated by triangles, i.e., each consecutive triplet of vertices defines a new triangle. A triangular grid is obtained. This triangular grid is important in the sense that it has a simple mathematical representation, it has several symmetry axes and a rotation of 90° will map the grid into itself. In addition, the triangle can be enlarged into similar shapes by using a step size parameter. This process will change the resolution of the images and will facilitate the fractal analysis. The number of triangles linked together will generate a new triangle shape that will influence the efficacy of surface area estimation. The sum of the areas of all the triangles within it will estimate the surface area.

According to the method presented in [21], a pre-defined fractal scaling parameter of size *k* = 1 is considered to build a matrix X_1_. In the next step, *k* = 2 and a similar triangle shape but one that is magnified twice will be considered, and a new matrix X_2_ is formed. This process is repeated until the fractal scaling parameter reaches *k* = 8. *k* = 1 means that all the points (*i*, *j*, $\gamma_{ij}$) of the analyzed surface are considered when approximating the surface *f*. Then, for *k* = 2 we increase the step size and repeat the process. We chose *k* = 8 so that the analysis still ensures informative details. In addition, this resolution has little effect on the error value of the calculated fractal dimension.

**Figure 4:**
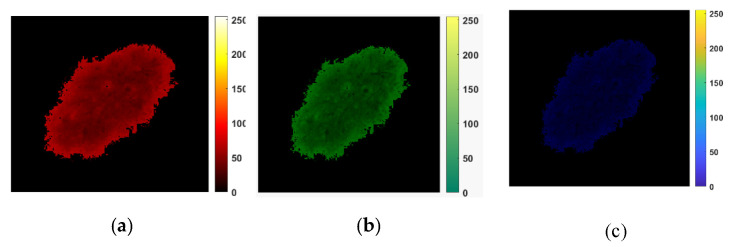

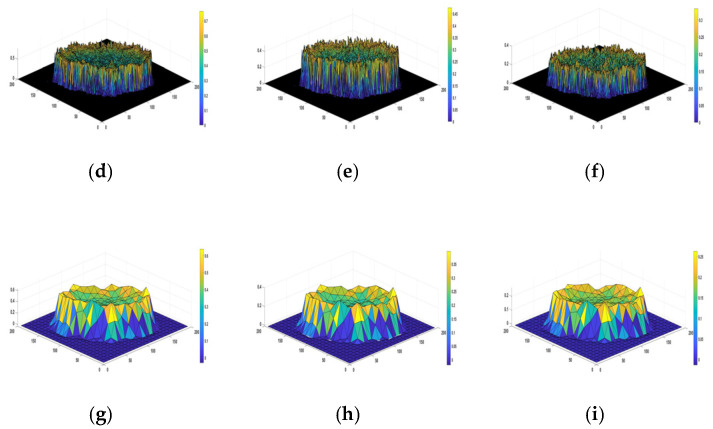
An example of the HFD computation. (**a**–**c**) R, G, and B color channels for a digital image that belongs to the PH2 dataset. (**d**–**i**) An illustration of the tessellation patterns for each color channel. The triangle shapes of *k* = 1 are presented in (**d**–**f**); the fractal scaling parameter *k* = 4 is shown in (**g**,**i**). They are used to compute the $X_{k_{c}}^{m}$ matrices. (**d**,**g**) are R channel images. (**e**,**h**) are G channel images. (**f**,**i**) are B channel images.

Figure 4 depicts a sample of results consisting of R, G, and B color channels in the first row and two triangle shapes (*k* = 1 and *k* = 4) for tessellation for each color channel in the last two rows. This intermediate step allows for the computation of the $\;X_{k_{c}}^{m}$ matrices and finally for 2D Higuchi’s surface fractal dimension, as discussed in this section.

An RGB image is separated in the R, G, and B color channels. Each color channel is analyzed as an intensity image. For each R, G, and B color channel, a $\;X_{k_{c}}^{m}$ matrix is formed as follows:

$$X_{k_{c}}^{m}=\left[\begin{array}{ccccc} \gamma_{m,m} & \gamma_{m+k_{{}_{c}},m} & \gamma_{m+2k_{{}_{c}},m} & \ldots & \gamma_{m+pk_{c},m}\\ \gamma_{m,m+k_{{}_{c}}} & \gamma_{m+k_{{}_{c}},m+k_{{}_{c}}} & \gamma_{m+2k_{{}_{c}},m+k_{{}_{c}}} & \ldots & \gamma_{m+pk_{c},m+pk_{c}}\\ \ldots & \ldots & \ldots & \ldots & \ldots \\ \gamma_{m,m+sk_{{}_{c}}} & \gamma_{m+k_{{}_{c}},m+sk_{{}_{c}}} & \gamma_{m+2k_{{}_{c}},m+sk_{{}_{c}}} & \ldots & \gamma_{m+pk_{{}_{c}},m+pk_{{}_{c}}} \end{array}\right]$$

where *k_c_* = 1, …, 8, and c denotes R, G, and B color channels. *m* = 1, 2, …, *k_c_*, $p=\left[\frac{M-m}{k_{c}}\right]$, $s=\left[\frac{N-m}{k_{c}}\right]$, and [] represents the integer part. 

The area of the surface *f* will be approximated as the sum over all the triangles $A_{m}\left(k_{c}\right)$, where c=R, G, B, whose vertices are given by the matrices $X_{k_{c}}^{m}$. By multiplying the differences of neighboring pixels in the triangle vertices, the area is estimated as follows [36]:

$$A_{m}\left(k_{c}\right)=\xi_{c}\sum_{i=1}^{\left[\frac{M-m}{k_{c}}\right]}\sum_{j=1}^{\left[\frac{N-m}{k_{c}}\right]}\left\{\begin{array}{l} \left|\gamma_{m+\left(i-1\right)k_{c},m+jk_{c}}-\gamma_{m+\left(i-1\right)k_{c},m+\left(j-1\right)k_{c}}\right|\cdot \\ \left|\gamma_{m+ik_{c},m+jk_{c}}-\gamma_{m+\left(i-1\right)k_{c},m+k_{c}}\right|\cdot \\ \left|\gamma_{m+ik_{c},m+jk_{c}}-\gamma_{m+ik_{c},m+\left(j-1\right)k_{c}}\right|\cdot \\ \left|\gamma_{m+ik_{c},m+\left(j-1\right)k_{c}}-\gamma_{m+\left(i-1\right)k_{c},m+\left(j-1\right)k_{c}}\right| \end{array}\right\}$$

where $\xi_{c}=\frac{1}{2k_{c}^{4}}\frac{\left(N-1\right)}{\left[\frac{N-m}{k_{c}}\right]}\frac{\left(M-1\right)}{\left[\frac{M-m}{k_{c}}\right]}$ is a normalization factor. Furthermore, the $A_{m}\left(k_{c}\right)$ were averaged for all *m* = 1, 2, …, *k_c_*. The average surface area $A\left(k_{c}\right)$ is:

$$A\left(k_{R}\right)=\frac{\sum_{m=1}^{k_{R}}A_{m}\left(k_{R}\right)}{k_{R}},A\left(k_{G}\right)=\frac{\sum_{m=1}^{k_{G}}A_{m}\left(k_{G}\right)}{k_{G}}\;\mathrm{and}\;A\left(k_{B}\right)=\frac{\sum_{m=1}^{k_{B}}A_{m}\left(k_{B}\right)}{k_{B}},\;kc=1,\;\ldots ,\;kmax.$$

Each collection of points plots a double logarithmic curve, $ln\left(A\left(k_{c}\right)\;\right)$ versus $ln\left(A\left(k_{C}^{2}\right)\;\right)$, that is fitted by the least squares linear method. The slope of the resulting best straight line, i.e., $S_{C}=ln\left(A\left(k_{C}\right)\;\right)/ln\left(A\left(k_{C}^{2}\right)\;\right)$ will allow for the computation of the Higuchi fractal dimension for each color channel as $HFD_{c}=S_{c}+1$. The average of $HFD_{c}$ values will provide the HFD feature. The 2D HFD values range from 2 to 3.

### 3.3. K-Nearest Neighbor (kNN) with 5-Fold cross Validation (kNN-CV) 

kNN is a machine learning algorithm that assumes that the samples of each group are predominantly surrounded by samples from the same group [38]. Usually, kNN has training and classification steps. The training step keeps the features and class label of the training samples. Then, kNN classifies new instances based on a similarity operation. The Euclidean distance is the most frequently used similarity measure. 

When only a small dataset is available, the cross validation technique is an alternative solution to the augmentation technique. In addition, the sampling cost is low. The kNN with a 5-fold cross validation (kNN-CV) algorithm uses a subset of data for validation but it still uses all the data for the testing phase. The input data is split into five folds. The algorithm is trained on four of the folds and tested on the single left-out fold. Then, this process is iteratively repeated until each fold becomes a test set and all data are evaluated. The training time complexity is O (*d* × *n* × log*n*), where the number of samples in the training dataset is denoted generically by *n* and data dimensionality by *d*. The complexity is an average. For 5-fold cross validation kNN, we repeat the computations and increase the time complexity. This relatively higher computation time could be interpreted as a limitation of the kNN-CV algorithm. This small drawback is compensated for by the reduction in the overfitting influence. Moreover, a correct estimation of the test error is its main advantage. It also provides a good level of accuracy for the model [39]. 

### 3.4. RBFNN Classifier

Artificial neural networks consist of a set of linked nodes that collaborate to solve problems. The RBF neural network is a three-layer feedforward neural network architecture. It is simple and fast and shows a very good tolerance to input noise. Each hidden node puts into action a nonlinear activation function, whicg is a radially symmetric function. The radial basis function is centered on a vector in the feature space and its response varies monotonically with distance from the central point. RBFNN can be formulated as the minimization of MSE function. A critical point for an RBF network implementation consists of the RBF centers and weights determination. The main advantages of the RBFNN are as follows: (i) it has a superior approximation ability for the interpolation operation, as it provides predictions in between the training set; (ii) it is a local approximation network as the outputs are provided by specified hidden neurons, and (iii) it uses hyper spheres to separate clusters. Thus, for an appropriately localized RBFNN, an input generates an important activation in a small region so that the issue of local minima is avoided [40,41]. These characteristics recommend the RBFNNs for modeling complex systems.

**Figure 5:**
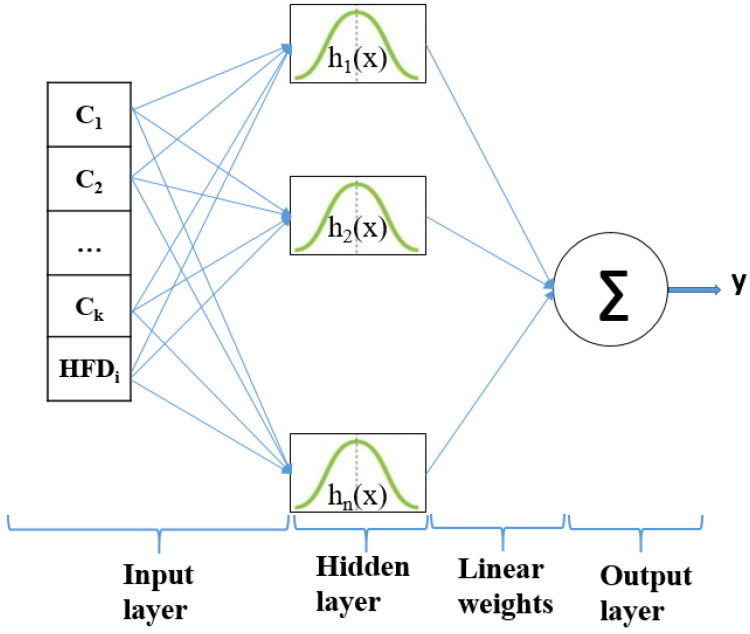
The structure of an RBFNN classifier.

Figure 5 depicts the RFB net architecture. The input layer allocates the $c_{k}+HFD_{i}$ features to the nodes of the hidden layer. $c_{k}$ is the average percentage of color area feature and $HFD_{i}$ is Higuchi’s fractal dimension for each dataset. The employed radially symmetric functions are Gaussian function $h_{1},\;h_{2},\;\ldots ,\;h_{n}$. The output layer consists of a summation over the number of possible output classes. The classifier prediction for an output class is the “spike” output over the summation.

Once the RBF centers and nonlinearities in the hidden layer are determined, the weights are computed based on the linear regression of the hidden layer outputs to the desired outputs or target values. The RBFNN classifier is trained and validated using 5-fold cross validation to strengthen the capabilities of the predictive model [38]. This approach separates the datasets into training and testing groups and avoids net overfitting.

### 3.5. Dataset Description

The proposed methodology was trained, validated, and tested on 248 nevi and 407 melanoma images collected from three databases, i.e., 7-Point (68 nevi/297 melanoma), PH2 (80 nevi/40 melanoma), and Med-Node (100 nevi/40 melanoma) [5,42,43]. The used classifiers do not require data augmentation techniques to mitigate the unbalanced data among the classes of skin lesions. The features (i.e., HFD and the average percentage of color areas/color clusters) were determined using a custom program written in Matlab (Matlab v. R2020b, Mathworks). Statistical analyses were completed using SPSS software (IBM SPSS Statistics 20). The hardware used was a computer with the following specifications: Inter (R) Core (TM) i7-8550U CPU @ 1.80 GHz; Memory (RAM) 8 GB DDR4; GeForce MX150 4 GB video; hard disk 500 GB SSD. Segmentation and features extraction had a processing time in the range of a couple of seconds. Overall, the processing time for each dataset was between 120 and 200 s.

A sound analysis based on the sensitivity, accuracy, precision, AUC (area under the curve), and Dice score metrics was used to determine the performance of the skin lesions classification.

## 4. Results and Discussion

The first step is image pre-processing for hair and noise removal and image segmentation. The second step performs the feature selections. The integration of color and fractal features in the analysis results in high feature dimensionality including a high level of feature redundancy. For reducing the redundant features in the feature space, the *t*-test is used to optimize the classification model. The selected features from each data set are shown in Table 1.

We separately tested and compared the efficacy of selected color clusters and fractal features by using a 5-fold cross validation and kNN classifier. We did not find any studies comparing classification performance of skin cancer diagnoses based on 2D Higuchi’s surface fractal features in the literature, so we compared the two classifiers in terms of accuracy.

**Figure 6:**
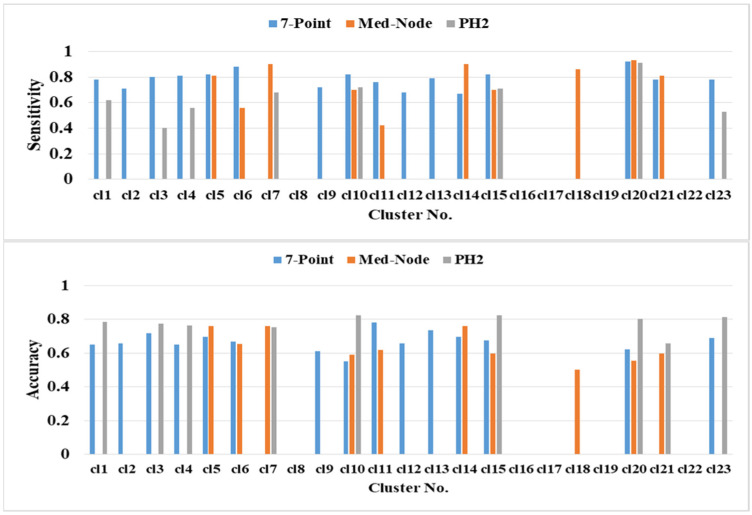

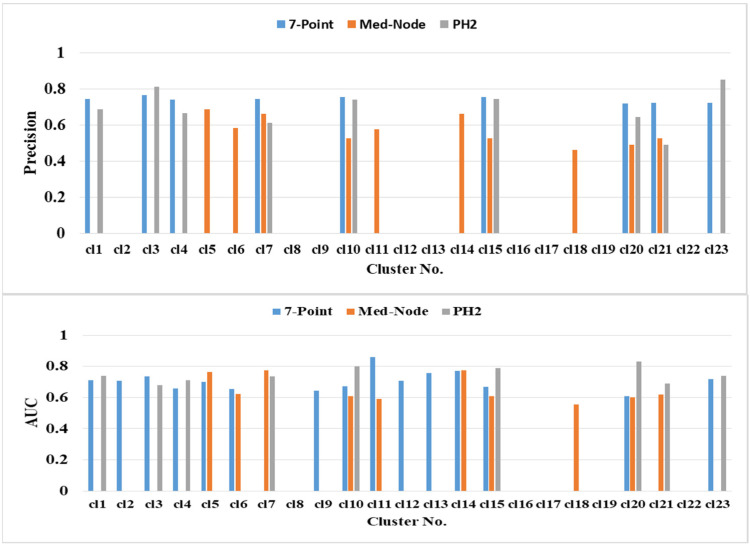
The prediction performance for the 5-fold cross validation and kNN classifier for different average percentage of color areas/color cluster descriptors.

The prediction performance of kNN based on the average percentage of the feature descriptors of colored areas differed significantly in terms of color clusters and datasets (Figure 6). The highest classification accuracies of 82.47% (clusters cl10 and cl15), 81.44% (cl23), and 80.41% (cl20) belong to the PH2 dataset. The second highest classification accuracy of 75.91% (cl5 cl7 and cl14) belongs to the Med-Node dataset. The color clusters cl3, cl11, and cl13 from the 7-Point dataset yielded lower accuracy results. The color clusters cl8, cl16, cl17, cl19, and cl22 did not contain any relevant average percentage of feature descriptors of color areas.

As introduced in Section 3.2, the 2D Higuchi’s surface fractal descriptor was computed for each image with the fractal scaling parameter varying from k = 1 to k = 8. The performance of classification based on the 2D Higuchi’s surface fractal descriptor is displayed in Table 2. Classification results show that the 2D Higuchi’s surface fractal descriptor produces a higher classification accuracy (79.38%) than the average percentage of feature descriptors of color areas for the PH2 dataset. 

Further, the selected discriminant features were classified by a 5-fold cross validation and RBFNN approach. The input data was split into five folds; one subset was the test set and the other four subsets were for training. The employed Gaussian functions *h*_1_, *h*_2_, …, *h_g_* were as follows: *g* = 15 for the 7-Point dataset; *g* = 11 for the Med-Node dataset, and *g* = 10 for the PH2 dataset.

The investigation was devoted to establishing the best performance for the RBFNN classifier with different inputs: 50 neurons on the hidden layer and two outputs. The number of hidden neurons varied from 0 to 50, and the new hidden nodes were automatically incorporated by the net. The experimental verification indicated that 50 neurons on the hidden layer assured the best classification performance and the classifier achieved the global optimal solution characterized by the lower MSE. Our specified mean squared error goal was 0.01. The diagnostic performance of the models was assessed in terms of sensitivity, accuracy, precision, AUC, Dice scores, and MSE provided by the RBFNN classifier. To highlight that both features can promote each other in the diagnostic process, the average percentage of color areas and HFD features were evaluated and compared together and separately, and the performance changes are presented in Table 3. 

Table 3 shows that for all datasets under investigation, when the input of the neural network consisted of both average percentage of color areas/color cluster and 2D Higuchi’s surface fractal descriptors, the diagnostic performance was improved in terms of accuracy, AUC, Dice scores, and MSE. In addition, the proposed RBFNN proved to be more accurate and efficient than the kNN algorithm in the recognition and classification of skin cancer. The RBFNN’s errors were smaller when input data were color clusters and HFD descriptors, confirming our working hypothesis of using the 2D Higuchi’s surface fractal descriptors to improve the performance of classification was correct. In addition, the skin lesions analysis was more precise when the proposed RBFNN was employed.

Traditionally, skin lesions are monitored in order to evaluate their type and evolution towards a potential cancerogenic pathology. Sometimes this is a lengthy process, and it can be quite stressful for the patient. In addition, as reported in the literature [44,45,46], melanomas and nevus may show different biological manifestations according to their anatomic locations (e.g., UV shielded sites and unshielded sites melanoma) and this can add to the complexity of the problem. The addressed lesion features in the proposed method could be used to investigate both of these kinds of cancerous cutaneous lesions. By using the proposed method, it is possible to quickly determine the skin lesions’ characteristics and establish the type of lesion, thus avoiding a lengthy monitoring process and assuring a correct and early diagnosis. In addition, the proposed solution is able to analyze various image types, provided by dermoscopy, clinical, histopathology, or confocal microscopy [46]. Current research [47,48] shows that several cases make diagnosis difficult, especially melanomas hidden by tattoos or atypical (or dysplastic) nevi [49]. In addition, the evolution of melanoma can include subtle changes that cannot be observed by a physician but can play an important role in establishing the type of skin lesion. The proposed surface fractal analysis overcomes this drawback.

To the best of our knowledge, 2D Higuchi’s surface fractal descriptors have not been previously used to recognize and classify nevus and melanomas in a neuronal network model. A comparative analysis to other reported results can only be done qualitatively with reference to the classification accuracy (Table 4). We compared our results to both machine learning and neural network methods. The classification accuracy of kNN-CV with 2D Higuchi’s surface fractal features is comparable to that provided by other classifiers. It can be noted that the proposed RBFNN algorithm achieved significant accuracy improvement in all cases.

There are some limitations of this study. The number of images was still limited despite using the cross-validation method. In addition, this study was devoted to only one method, 2D Higuchi’s surface fractal computation. To overcome this limitation, other methods for determination of 2D fractal dimensions are possible. However, the decorrelated features, such as color clusters and HFD, utilized together in the classification task allow for an accurate classification using a relatively small number of images. 

## 5. Conclusions

In this paper, a study based on the skin surface fractal dimensions and relevant average percentages of the color area features was conducted for skin lesion classification. The results indicated that appropriately generated color features that were further correlated to fractal features allowed the RBFNN classifier to provide high accuracies of classification. 

To the best of our knowledge, 2D Higuchi’s surface fractal features have not been previously used for skin lesion classifications. In future work, other image features will be considered together with the surface fractal features. In addition, in order to improve the classification accuracy, a more balanced dataset will be considered (either by populating it with an increased number of images or by using augmentation methods).

## Figures and Tables

**Table 1:** Selected features and their numbers used in the experimental analysis.

Dataset	Color Cluster Features	2D Higuchi’s Surface Fractal
7-Point	15 average percentage of color areas: cl1, cl2, cl3, cl4, cl5, cl6, cl9, cl10, cl11, cl12, cl13 cl14, cl15, cl20, cl23 5475 features	365 features
Med-Node	10 average percentage of color areas: cl5, cl6, cl7, cl10, cl11, cl14, cl15, cl18, cl20, cl21 1400 features	140 features
PH2	9 average percentage of color areas: cl1, cl3, cl4, cl7, cl10, cl15, cl20, cl21, cl23 1080 features	120 features

**Table 2:** Accuracy for 5-fold cross validation and kNN classifier for 2D Higuchi’s surface fractal descriptors.

Dataset	Sensitivity (%)	Accuracy (%)	Precision (%)	AUC	Dice Score
7-Point	80.77	71.43	73.26	0.6948	0.7683
Med-Node	30.19	64.23	57.14	0.6423	0.3951
PH2	83.33	79.38	62.50	0.8047	0.7143

**Table 3:** Performance of the RBFNN classifier in the testing experiments.

Dataset	RBFNN Inputs	Sensitivity (%)	Accuracy (%)	Precision (%)	AUC	Dice Scores	No. of Hidden Neurons	MSE
7-Point	Color clusters	97.77	95.12	94.32	0.9412	0.9603	50	0.1904
	Color clusters and HFD	98.01	95.42	94.44	0.9422	0.9630		0.0924
Med-Node	Color clusters	96.22	94.12	88.61	0.9550	0.9333	50	0.1789
	Color clusters and HFD	96.42	94.71	87.50	0.9588	0.9396		0.1662
PH2	Color clusters	1.00	94.17	85.03	0.9553	0.9195	50	0.1372
	Color clusters and HFD	1.00	94.88	85.62	0.9685	0.9211		0.1128

**Table 4:** The comparison of accuracy results of the proposed method with those of existing methods.

Authors	Accuracy (%) and Details
Nasiri et al. [50]	64% (for 1st test: kNN (300, 100) and spot features)
	67% (2nd test: kNN (1346, 450) and spot features)
Kavitha et al. [51]	78.2 (kNN and GLCM features)
Al-masni et al. [52]	81.79% (Inception-ResNet-v2, ISIC 2016 dataset)
	81.57% (ResNet-50, ISIC 2017 dataset)
	89.29% (ResNet-50, ISIC 2018 dataset)
Seeja & Suresh [53]	79.26% (kNN, LBP and Edge histograms, HOG, Gabor filter)
Khan et al. [54]	94.50% (Neural Network/Feed Forward/sigmoid function/3 hidden layers, ISBI2016 dataset, 70:30 training and testing).
	94.20% (Neural Network/Feed Forward/sigmoid function/3 hidden layers, ISBI2017 dataset, 70:30 training and testing).
Proposed kNN-CV	71.43% (7-Point dataset); 64.23% (Med-Node dataset) and 79.38% (PH2 dataset) for 2D Higuchi’s surface fractal features
Proposed (RBFNN—color clusters and HFD)	95.42% (7-Point dataset)
	94.71% (Med-Node dataset)
	94.88% (PH2 dataset)
